# Label-free analytic histology of carotid atherosclerosis by mid-infrared optoacoustic microscopy

**DOI:** 10.1016/j.pacs.2022.100354

**Published:** 2022-04-11

**Authors:** Mirjam Visscher, Miguel A. Pleitez, Kim Van Gaalen, Ingeborg M. Nieuwenhuizen-Bakker, Vasilis Ntziachristos, Gijs Van Soest

**Affiliations:** a Department of Cardiology, Erasmus MC University Medical Center Rotterdam, PO Box 2040, 3000 CA Rotterdam, The Netherlands; b Institute of Biological and Medical Imaging, Helmholtz Zentrum München, Neuherberg, Germany; c Chair of Biological Imaging (CBI) and Center for Translational Cancer Research (TranslaTUM), Technische Universität München, München, Germany

**Keywords:** Mid-infrared optoacoustic microscopy, Atherosclerosis, Spectroscopy, Cholesterol, Pathology

## Abstract

**Background and aims:**

Analysis of atherosclerotic plaque composition is a vital tool for unraveling the pathological metabolic processes that contribute to plaque growth.

**Methods:**

We visualize the constitution of human carotid plaques by mid-infrared optoacoustic microscopy (MiROM), a method for label-free analytic histology that requires minimal tissue preparation, rapidly yielding large field-of-view en-face images with a resolution of a few micrometers. We imaged endarterectomy specimens (n = 3, 12 sections total) at specific vibrational modes, targeting carbohydrates, lipids and proteins. Additionally, we recorded spectra at selected tissue locations. We identified correlations in the variability in this high-dimensional data set using non-negative matrix factorization (NMF).

**Results:**

We visualized high-risk plaque features with molecular assignment. Consistent NMF components relate to different dominant tissue constituents, dominated by lipids, proteins, and cholesterol and carbohydrates respectively.

**Conclusions:**

These results introduce MiROM as an innovative, stain-free, analytic histology technology for the biochemical characterization of complex human vascular pathology.

## Highlights

• Highly heterogeneous atherosclerotic plaque can be characterized by mid-IR optoacoustic microscopy.
• Images at salient wavelengths show different lipids and protein structures.
• Three spectrocopically distinct tissue types could be identified in advanced plaque by unsupervised clustering.

## 1 Introduction

Cardiovascular diseases are the leading cause of death, taking almost 18 million lives worldwide in 2019 [1]. Of these, 85% are caused by heart attacks and stroke, conditions for which atherosclerosis is the underlying substrate in the majority of cases. This makes atherosclerosis the single largest cause of morbidity and mortality worldwide. The formation of atherosclerosis remains a topic of intense study, as changing demographics and lifestyle patterns affect the disease’s phenomenology and impact [2].

Highly heterogeneous plaques form in the arterial wall as a result of inflammation-driven deposition of lipids. Initiated by the infiltration of low density lipoproteins (LDL) into the sub-endothelial layer [3], lipids play a major role in the development of atherosclerosis. Analysis of plaques with an unstable phenotype showed elevated levels of cholesterol and cholesteryl ester (CE) species [4]. Such plaques exhibit high inflammation activity, necrosis, impaired connective matrix, and low smooth muscle cell (SMC) content. Formation of cholesterol crystals (CC) is a driver of plaque destabilization and inflammation [5], [6], induces apoptosis of macrophages [7], and may impact the cell membrane of macrophages [8]. It is clinically associated with ischemic events [9]. Solid cholesterol in atherosclerosis has been described as needle-shaped, plate-shaped, filamentous or helical form [10]. Seemingly stable plaques, which are more homogeneous with increased organized collagen and SMCs, exhibit a higher abundance of phospholipids (PL) and triacylglycerides [4]. The mechanisms that lead to this variance in lipidomic appearance, the interactions with local metabolic and enzymatic activity, and modification of these relations by pharmacological interventions, remain largely unknown. Molecular characterization of the native biochemistry of atherosclerosis therefore is important for understanding plaque formation.

Imaging remains a crucial tool in (molecular) pathology studies of atherosclerosis. In histochemical assays, the current standard for lipid is the Oil Red O stain. This stain is class unspecific, and only binds to neutral lipid species in droplet form, excluding biologically relevant species like phospholipids, sphingolipids, ceramides and crystalline cholesterol. In conventional histochemistry, CC is only detectable by absence as unstained cleft- or needle-shaped voids in the tissue section [10], since common histochemical stains do not stain CC, or cholesterol is dissolved during tissue washing steps. As with any form of histological staining, detailed tissue handling and processing protocols need to be observed. Spatial lipid distribution in atherosclerosis, and associations of localized CE, sphingomyelin (SM) and lysophospholipids with high-risk plaque features, were studied by mass spectrometry imaging (MSI), a label-free molecular imaging technique. Tissues that are homogeneous by histological classification exhibit a large variability in lipid content [11]. Limitations in MSI are a strong dependence of the chemical sensitivity on experimental parameters, long scan times, and expensive equipment, which inhibit routine imaging of excised tissue. Specific detection of cholesterol has been challenging using MSI. This demonstrates the need for additional and more accessible chemically specific imaging to further disentangle the etiology of atherosclerosis.

The chemical specificity of vibrational spectroscopy in the mid-infrared (mid-IR) spectral region (4000–400 cm^−1^) allows the imaging of endogenous biomolecules by techniques such as mid-IR absorption and Raman Scattering microscopy, adding chemical specificity to histology. The mid-IR range is conventionally divided in the so-called fingerprint (FP) and CH or high-wavenumber regions. The CH region (4000–2800 cm^−1^) is dominated by fundamental C—H vibrations, while the FP region (approximately 1800–400 cm^−1^) is populated by complex molecular vibrational patterns. Mid-IR and (non-linear) Raman imaging of atherosclerosis [12], [13], [14], [15] yielded a clear distinction between CC and condensed CE structures in intact plaques [15], characterization of vulnerable plaque features such as the fibrous cap, calcification and lipid changes in the necrotic core [13], and lipid distribution in the sub-endothelial space of the intima [14].

Mid-IR absorption spectroscopy is label-free and provides micrometer-range imaging resolution of several molecular species in a single experiment, but has limited sensitivity due to the negative-contrast detection approach (i.e., the larger the optical absorption, the weaker the signal detected) of conventional optical spectroscopy. In this study we applied mid-infrared optoacoustic microscopy (MiROM) [16], to image the molecular composition of advanced carotid atherosclerotic plaques. MiROM shares the benefits of diffraction-limited spatial resolution and high spectral resolution of conventional mid-IR microspectroscopy, but offers high sensitivity as it is based on positive-contrast detection (i.e., the larger the absorption, the stronger the signal detected). MiROM was previously applied to living cells and animal tissues [17], but not yet for the detailed biochemical characterization of human pathology.

Advanced atherosclerotic plaques present a highly complex and variable mix of lipids, proteins, carbohydrates and other molecules. Their composition reflects local metabolic and cellular processes, and MiROM offers a window onto plaque biochemistry of intact, unstained specimens. In this study we imaged whole human carotid endarterectomy gross transversal sections (n = 12) at selected vibrational modes, with multi-stain classical histochemical analysis as reference. Additionally, we acquired images at selected regions-of-interest and performed full spectral scans at sites selected based on heterogeneity and presence of high-risk features. With these data, we illustrate the use of MiROM as an analytical histology tool for assessing intrinsic lipid, glycan and protein distribution in advanced human atherosclerotic plaque.

## 2 Materials and methods

### 2.1 Tissue collection and processing

Three human carotid endarterectomy (CEA) plaque samples were surgically harvested and were washed in PBS, snap frozen and stored at − 80 °C until further processing. A special surgical protocol was performed to preserve an intact lumen and morphology of the specimen [18]. CEA samples were divided into 2 mm thick cross-sections and embedded in 10% porcine gelatin type-A (Sigma-Aldrich, The Netherlands). The tissues were cryo-sectioned (CM3050 S, Leica Biosystems; cutting temperatures: OT −21 °C; CT −19 °C) into 10 µm thick sections, thaw mounted onto glass slides and stored at − 80 °C. The remaining tissue block, 1–1.5 mm in thickness, was used for MiROM microscopy. Twelve blocks were included in this study. This study was performed according to the ethical guidelines sanctioned by the Ethics Board of Erasmus MC (MEC 2008–147).

### 2.2 Histology, histology segmentation and image registration

The tissue sections were histochemically stained by: Hematoxylin and eosin stain (HE), Miller’s elastic stain, Martius scarlet blue trichrome (MSB), Oil Red O (ORO) and Periodic acid–Schiff (PAS) combined with Alcian blue (AB), to stain for general structures, collagen and elastin, fibrin and erythrocytes, lipids, and polysaccharides, respectively.

Based on the histological information the tissue section was segmented (MevisLab v2.7.1) into the following plaque components; necrotic core (NC), fibrin, foam cells (FC), erythrocytes, calcium and cholesterol crystals (Suppl. Fig. S1).

### 2.3 Mid-infrared optoacoustic microscopy

The Mid-infraRed Optoacoustic Microscopy (MiROM) system used here, is reported and explained in detail in Ref. [16]. Briefly, a tunable pulsed quantum cascade laser (QCL) (MIRcat, Daylight Solutions) generates an optoacoustic signal in tissue, which is detected in transmission-mode using a focused 20 MHz ultrasound transducer (Imasonic). The spectral range of the QCL is 3.41–3.61 µm (2932–2770 cm^−1^) and 5.75–11.11 µm (1739–900 cm^−1^) with a line width of ≤ 1 cm^−1^ (pulse duration 20 ns; repetition rate 100 kHz). The laser is focused using a 0.5 NA gold-coated reflective objective (36 ×, Newport Corporation), confocally-aligned with the ultrasound transducer. The raw optoacoustic signal was amplified by 63 dB (MITEQ), low-pass filtered (cut-off 50 MHz; Mini-Circuits), and digitized by a data acquisition system (Gage Applied) at a sampling rate of 250 MSs^−1^. The images show the peak-to-peak amplitude of 50 averaged optoacoustic pulses. In the study presented here, images were obtained by raster-scanning the sample using motorized stages (Physik Instrumente). The imaging speed obtained with this method is around 240 pixels/sec (or 3.8 ms/per pixel). For instance, MiROM enables imaging a FOVs of 5 mm × 5 mm in steps of 10 µm (pixel size) in 16 min for each excitation wavelength.

**Fig. 1:**
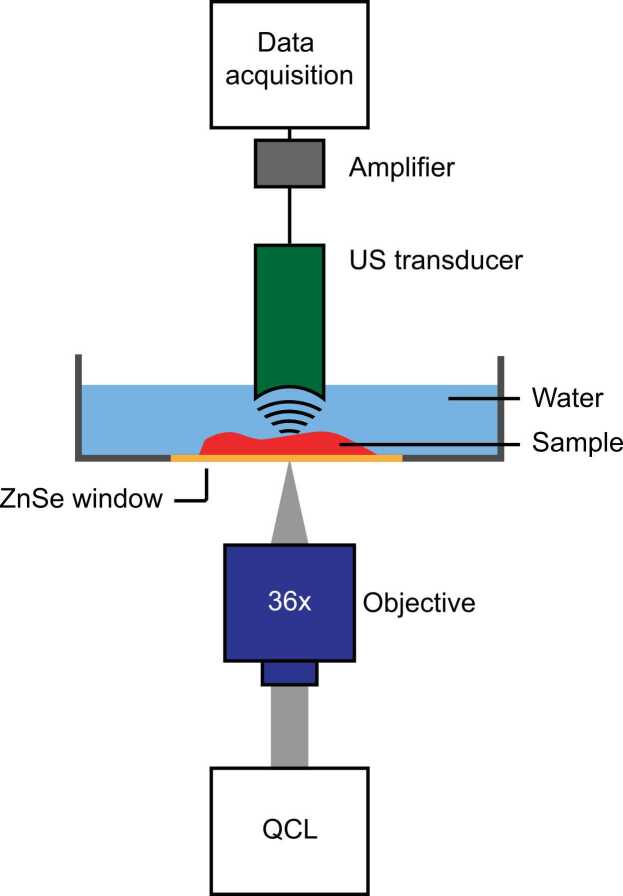
Schematic of the MiROM microscope.

The tissue block was thawed and placed onto a custom-made sample holder with a mid-IR transparent ZnSe window (Edmund Optics). After desiccation under vacuum for 10 min to remove bubbles between the sample and the window, the sample was covered with acoustic-transparent film and immersed in deionized water for acoustic coupling. A schematic of the microscope setup is depicted in Fig. 1.

Images of the full field of view of the CEA samples were recorded, at wavelengths characteristic of documented vibrational features [14], [19], [20], with a scanning step size of 25 µm. Per cross-section, one or more regions of interest were additionally selected and imaged at higher spatial resolution, 2.5 or 5 µm. At specific locations, absorption spectra were recorded with a spectral step size of 2 cm^−1^ or 4 cm^−1^, averaging 10,000 traces per wavelength, throughout the CH (2941 – 2780 cm^−1^) and FP spectral regions (1739 – 909 cm^−1^). Acquired spectra were normalized to a reference signal from carbon tape to equalize laser output spectrum as well as to compensate for slight differences in OA signal intensity due to acoustic-detector alignment on different days. Images were then processed by contrast enhancement to 0.3% saturation, histogram normalization, bi-cubic interpolation and convolution with a 2-pixel-wide Gaussian filter.

### 2.4 Cluster analysis

We performed unsupervised clustering analysis by means of non-negative matrix factorization (NMF), using an NMF toolbox for biological datamining [21], [22] implemented in MATLAB 2019b (Natick, MA, USA). NMF in the spectral domain identified co-occurring patterns in all full-range spectra acquired at selected sites of interest. In the image domain, NMF clusters features in images acquired at six salient wavelengths. In both analyses, the optimum number of components was determined based on the dispersion coefficient [23].

## 3 Results

### 3.1 MiROM imaging

**Fig. 2:**
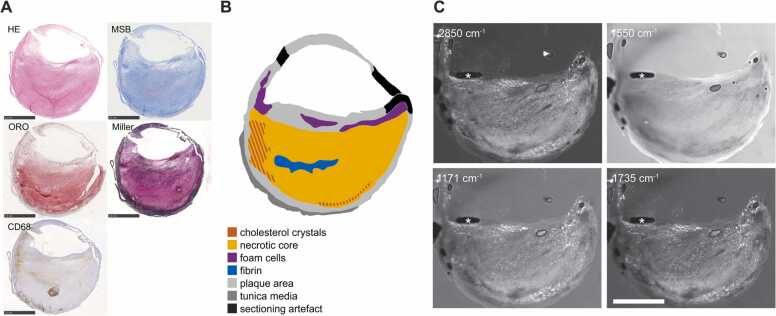
Histology, tissue composition and MiROM. (A) Five histological stains of an advanced CEA specimen. (B) Tissue type assignment based on histology. (C) fFOV MiROM images showing high signal for both 1550 cm^−1^ (protein) and various wavelengths associated with lipids, consistent with the histological assessment of lipid-rich, collagen-rich plaque. HE: Hematoxylin and Eosin; MSB: Martius scarlet blue; ORO: Oil Red O; * : coupling artefact. (For interpretation of the references to color in this figure legend, the reader is referred to the web version of this article.)

Twelve carotid endarterectomy (CEA) cross sections, originating from three plaques of three different patients, were imaged using the MiROM system. The histology, plaque type annotation and acquired MiROM data are illustrated by an example of a lipid-rich plaque with a matrix consisting of largely degraded collagen and elastin fibers, inflammation and fibrin deposits in Fig. 2. Full field of view (fFOV) MiROM images are included of Spectral peaks were assigned to vibrational modes, based on literature review of Raman and FTIR measurements of atherosclerotic tissues [14], [19], [20], see Table 1. The signal at 2850 cm^−1^ originates mainly from the symmetric stretching of CH_2_, which, though not exclusive of lipids, is stronger for lipids than for other biomolecules [24]. Wavelengths relating to the same class of molecules (such as 1171 cm^−1^ and 1735 cm^−1^ for CE, Fig. 2C) appear highly similar. The signal at 1550 cm^−1^ is introduced by amide II bonds, mainly present in proteins. Images at this wavelength are relatively featureless compared to those representing lipid features. Comparison with histology was used to corroborate these assignments; for example, similar patterns in the ORO staining and the 2850 cm^−1^ images (Suppl. Fig. S2) support the association between this wavelength and lipids.

**Table 1:** Overview of vibrational modes and wavelength at which they occur in MiROM with tentative molecular assignments based on literature [14], [19], [20].

Wavelength (cm^−1^; ± 2)	Vibrational mode	Tentative molecular assignment
2850	CH_2_ symmetric stretching	Lipids
2832		Cholesterol
1735	C=O stretching	Cholesteryl esters and triacylglycerols
1550	Amide II (C=N and N-H stretch)	Protein
1465	CH_2_ asymmetric scissoring	Lipids
1171	C-O-C asymmetric stretching	Cholesteryl esters
1085	PO_2_^-^ symmetric stretching C-O stretching	Phospholipids and nucleic acids Glycogen, oligosaccharides and glycolipids
1053	C-C stretching	Cholesterol and carbohydrates

**Fig. 3:**
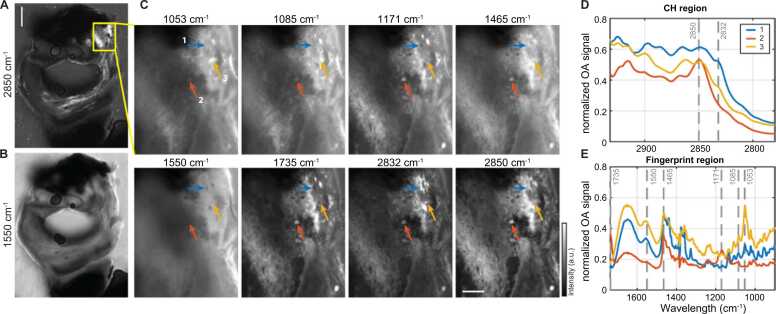
Example of MiROM measurement of CEA cross-section. fFOV MiROM image of cross-section at (A) 2850 cm^−1^, distribution of lipids, and (B) 1550 cm^−1^, protein. Scale bar in (A) 1 mm; yellow box delineates ROI shown in (C). (C) Zoomed MiROM ROI with arrows marking locations where spectra in (D,E) were recorded, scale bar 250 µm. (D) MiROM spectrum of CH region with vibrational bands at 2850 cm^−1^ (CH_2_ symmetric stretching) and 2832 cm^−1^ (shoulder peak of cholesterol). E) MiROM spectrum of FP region with vibrational bands at 1735 cm^−1^ (CH_2_ scissoring), 1550 cm^−1^ (Amide II), 1465 cm^−1^ (CH_2_ scissoring), 1171 cm^−1^ (C-O-C asymmetric stretching), 1085 cm^−1^ (C-O deformation), 1053 cm^−1^ (C-C stretching).

Fig. 3(A,B) depict the fFOV MiROM recorded maximum intensity projections of a heterogeneous, partially calcified, example CEA cross-section at 2850 cm^−1^ (lipids) and 1550 cm^−1^ (protein) and zoomed regions of interest (ROIs), where features of advanced or complex plaque were identified in the fFOV scans. In the ROIs, we recorded high-resolution (2.5 µm) images (16 ROIs), at a range of relevant wavelengths based on prior review of spectral features, Fig. 3(C). At selected locations in those zoomed images, we acquired full-scan spectra (37 sites); examples shown in Fig. 3(D,E).

### 3.2 Spectral analysis

**Fig. 4:**
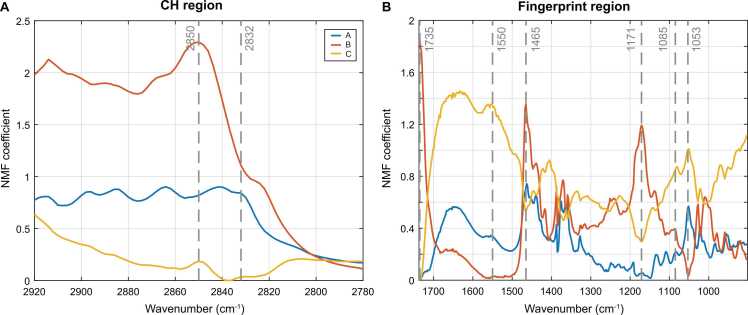
Non-negative matrix factorization of MiROM spectra results in 3 NMF components labeled A (blue), B (orange) and C (yellow). (A) NMF spectral decomposition of CH region and (B) NMF spectral decomposition of fingerprint region. Gray vertical lines depict the tentative molecular vibrational assignments, see Table 1. (For interpretation of the references to color in this figure legend, the reader is referred to the web version of this article.)

All available full-scan spectra (n = 37) were grouped and clustered using non-negative matrix factorization. This resulted in 3 NMF components, depicted in Fig. 4. Component A shows a correlation between the peak at 2832 cm^−1^ and 1053 cm^−1^, however the peak at 1053 cm^−1^ is also present in component C, combined with peaks of 1085 cm^−1^, 1550 cm^−1^. Component B has strong peaks at 2850 cm^−1^, 1735 cm^−1^, 1465 cm^−1^ and 1171 cm^−1^, all peaks that are tentatively assigned to vibrational modes dominated by lipids. Component C is strong in the FP region but low in the CH region, suggesting low correlation of this component to lipids.

**Fig. 5:**
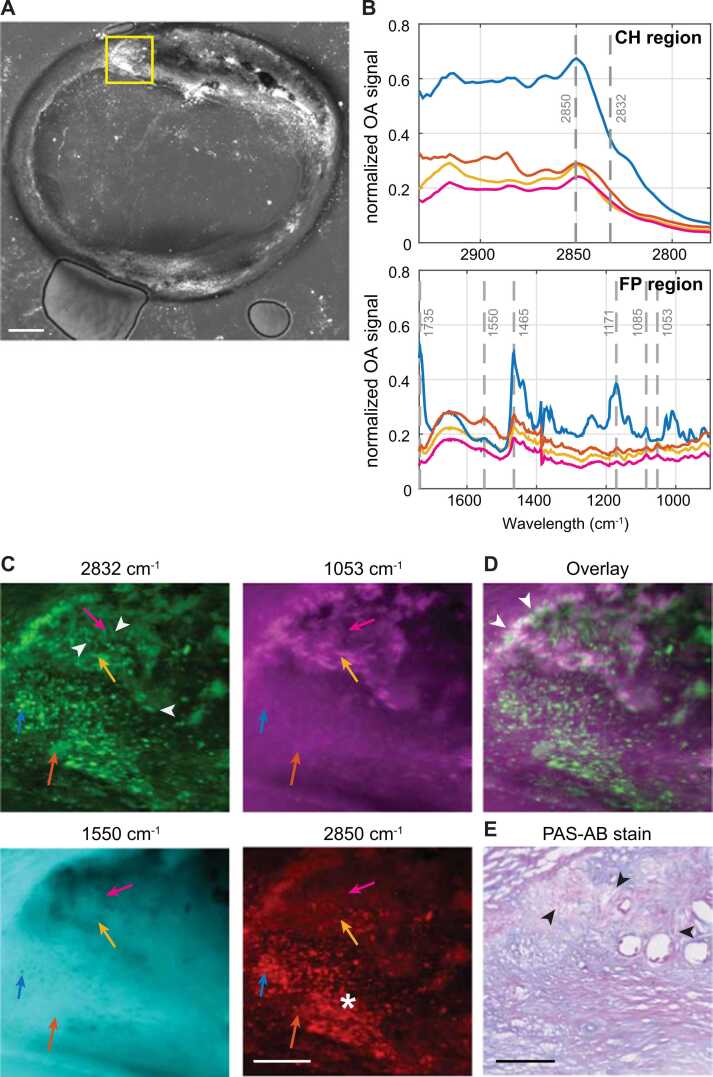
MiROM images showing lipid, protein, cholesterol and carbohydrate features. (A) MiROM image at 2850 cm^−1^; scalebar 500 µm. (B) CH- and FP-region spectra at selected locations (colored arrows) in (C). (C) Images at 2832 cm^−1^, 1053 cm^−1^, 1550 cm^−1^ (protein), and 2850 cm^−1^ (lipids); arrowheads: cholesterol crystals; * : lipid droplets. (D) Overlay of 1053 cm^−1^ (magenta) and 2832 cm^−1^ (green); arrowheads indicate overlapping signals. (E) Periodic acid-Schiff (PAS) and Alcian Blue (AB) histology, showing carbohydrate macromolecules like glycogen and glycolipids (PAS, stained purple) and acidic mucosubstances (AB, stained blue). Arrowheads: cholesterol crystal clefts. Scalebars in (C,E) are 250 µm. (For interpretation of the references to color in this figure legend, the reader is referred to the web version of this article.).

**Fig. 6:**
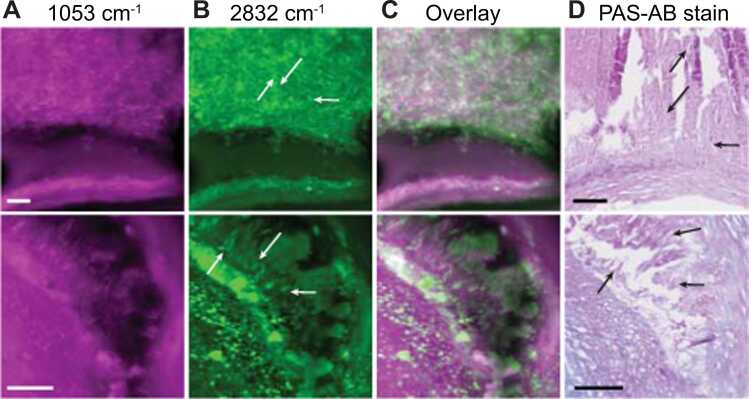
Zoomed MiROM images in two sections showing finely distributed cholesterol needles (top row) and sheets (bottom row). (A) MiROM images at 1053 cm^−1^, C-C stretching of cholesterol and carbohydrates. (B) Images at 2832 cm^−1^, shoulder peak of cholesterol with arrows indicating cholesterol crystals; overlaid in (C). (D) Combined Periodic acid-Schiff (PAS) and Alcian Blue (AB) staining of tissue, showing the presence of carbohydrate macromolecules like glycogen and glycolipids (PAS, stained purple) and acidic mucosubstances (AB, stained blue). All scalebars are 250 µm. (For interpretation of the references to color in this figure legend, the reader is referred to the web version of this article.).

Spectral NMF component A showed a correlation between 2832 cm^−1^ and 1053 cm^−1^, which is visible in the imaging data as co-localization of the two signals. Needle shaped structures are visible at both 1053 cm^−1^ and 2832 cm^−1^, see Figs. 5(C,D) and 6 (top row). Adjacent histological sections confirm that 2832 cm^−1^ can likely be attributed to cholesterol, and needle structures correspond to the presence of clefts. The PAS-AB stain shows more intense staining in the region around these clefts, revealing the presence of carbohydrates in those areas, see Fig. 5(E).

The peak at 1053 cm^−1^ could originate from a combination of the C-O stretching of the OH group in carbohydrates [19], [25] and C-O bending of Cholesterol [14], [25], [26]. Congruently, we observed that the contrast of images obtained at 1053 cm^−1^ wavelength partially overlaps with images obtained at 2832 cm^−1^ from cholesterol structures at the protein-poor necrotic core edge (Fig. 5(D), white arrows). The signal at 1550 cm^−1^, associated with protein, is low in the necrotic area.

Furthermore, cholesterol sheets, frequently found in atherosclerotic plaques [27] but not routinely identified in conventional histology, are visible at 2832 cm^−1^ as shown in Fig. 6 (bottom row). Interestingly, although lipid droplets can be identified both at 2850 cm^−1^ and 2832 cm^−1^ (Fig. 5(C), asterisk), the needle-shaped structures are predominantly observed in MiROM micrographs obtained at 2832 cm^−1^ (arrowheads), which supports the association with cholesterol that occurs predominantly in cleft-like structures.

### 3.3 Spatial cluster analysis

**Fig. 7:**
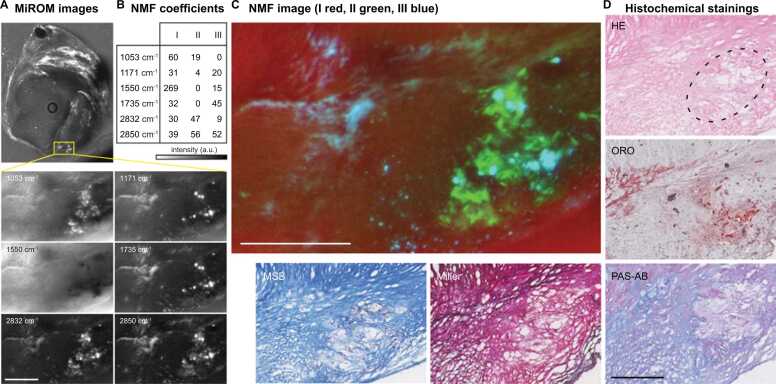
Example of MiROM images analyzed using NMF. (A) MiROM image of cross-section at 2850 cm^−1^, ROI indicated in yellow. ROI images at 1053, 1171, 1550, 1735, 2832, and 2850 cm^−1^, showing differences in spatial distribution between vibrational modes. (B) NMF weights of 6 vibrational modes. (C) Color image of 3 NMF components mapped to R-G-B (I-II-III) channels. (D) Histochemical stains of adjacent cross-sections, showing HE (general structure; dashed line: necrotic area), ORO (neutral lipids), PAS-AB (polysaccharides), Miller (elastin and collagen), and MSB (fibrin, erythrocytes and collagen). Scalebars are 250 µm. (For interpretation of the references to color in this figure legend, the reader is referred to the web version of this article.)

NMF was used to decompose six-wavelength MiROM images obtained at nine zoomed ROIs (from seven fFOV scans distributed over the three specimens; 54 images total). This analysis also resulted into 3 components, labeled in Roman numerals, see Fig. 7 for an example of this clustering applied to one of the zoom regions. Component I (red) is dominated by the protein band at 1550 cm^−1^ and is largely homogeneous, with generally lower intensity in the necrotic part and high intensity in collagen-rich areas as assessed by histology. Component II (green) displays contributions from 1053, 1171, 2832 and 2850 cm^−1^ and is co-localized with the necrotic area shown in histological staining. Component III (blue) reveals contributions from 1171, 1550, 1735 and 2850 cm^−1^, and resembles the ORO stain in Fig. 7(D), meaning it is most likely dominated by lipid signals and includes signals attributed to CE. Compared to component III, component II has a significantly higher contribution from 1053 and 2832 cm^−1^.

## 4 Discussion

With this study, we introduced MiROM for imaging the spatial distribution of different types of molecules in human atherosclerosis. With minimal tissue processing and scan times that can in principle be reduced to minutes per cross-section, MiROM imaged the spatial distribution of different vibrational modes, identifying the distribution of cholesterol crystals and sheets, overall lipid content, proteins and carbohydrates in plaques. We decomposed spectral and imaging information by means of NMF, each resulting in three distinct components that relate to dominant tissue components assessed by classical histopathology.

Cholesterol crystals are commonly found in atherosclerotic plaques and are considered an important marker, associated with an increased risk of plaque rupture, local inflammation, and occurrence of ischemic events [9], [10]. With MiROM spectra and images, we confirm the presence of cholesterol crystalline structures by the observation of a signal from vibrational modes associated with cholesterol directly, where traditional histology relies on the interpretation of empty cleft-like structures. In their direct vicinity, supported by PAS-AB staining, our observations suggest the presence of carbohydrate accumulation; potentially from glycated structures.

NMF analysis of the spectral data shows a clustering of lipid related vibrational modes, with contributions from 1171, 1465, 1735 and 2850 cm^−1^, in component B. Furthermore, component A shows a link between 1053, 1465, 1550 and 2832 cm^−1^. Component C shows a relation between 1053 and 1550 cm^−1^; this correlation supports the possibility that glycoproteins generate at least part of the signal at 1053 cm^−1^. A similar grouping of spectral features can be recognized in the spatial cluster analysis: the relative NMF weights of spatial cluster I resemble coefficient amplitudes of spectral component C, as II displays similarities with A and III with B.

This work represents the first study of MiROM applied to human pathological tissue. It demonstrates the utility in unraveling some of the chemical complexity and may be refined to identify specific features in atherosclerosis. The technique may prove similarly insightful in neurodegenerative diseases or cancer, where deposition of aberrant tissue leads to disease and even death. Mid-IR spectroscopic imaging studies of tissue composition in atherosclerosis have been reported previously [12], [13], [14], [24], [28], [29], [30]. Stimulated Raman Scattering imaged structures that could be identified as CC [31]. In contrast with previous works, where multivariate analysis of FT-IR microscopy and ATR-FT-IR spectra could not differentiate between normal and atherosclerotic rabbit aorta [28], our data shows a relation between spectral signatures and features of pathology. Fig. 7 shows distinct spatial patterns in the plaque by NMF analysis, which is unlike earlier hierarchical cluster analysis of ApoE/LDLR^-/-^ mouse aorta images, that did not find clustering within the plaque itself [14].

### 4.1 Limitations

In this study, vibrational modes are assigned to molecular composition based on literature review, and where possible validated using patterns observed in histopathology. The assignment is not unambiguous, and further research is necessary to validate our tentative assignments. However, validation hereof is complex because of the heterogeneity of the tissue.

In these proof-of-concept experiments we applied moving stage scanning, which is relatively slow. For clinical application, MiROM could be further enhanced for high-speed imaging by using fast galvo-mirror beam-scanning in combination with laser pulse excitation at high repetition rates (>1 MHz) as well as by improving the current scanning algorithm.

While our data set, limited to only 12 sections, represents the majority of tissue types commonly encountered in advanced carotid atherosclerosis, calcification was relatively scarce in the tissues we investigated. Large calcifications severely complicate histologic processing and are likely to introduce artifacts in MiROM, because the sectioned surface will typically not be flat. The uneven coupling that results from variable contact and the acoustic heterogeneity of the tissue cause signal dropout, see Suppl. Fig. S2 (P1, second image). A number of calcium specific spectral features related to (hydroxy)apatites has been identified in previous IR studies [26], which we could thus not replicate.

## 5 Conclusions

In conclusion, we have characterized twelve human carotid endarterectomy samples by means of MiROM imaging at a spatial resolution of 25 µm. In these sections, high-resolution (2.5 or 5 µm) images of 16 ROIs were recorded and spectral information was collected at 37 locations in these plaques. Comparison with histochemical analysis of adjacent cross-sections revealed corresponding patterns in specific tissue constituents and vibrational molecular modes in these areas. We performed unsupervised machine classification of the spectroscopic image data, revealing a consistent set of three components that represent salient features of atherosclerosis, such as necrosis, lipid-rich and collagenous tissue. Requiring minimal tissue processing, MiROM may become a valuable tool for label-free analytic histology for the study of atherosclerotic specimens.

## Financial support

This work is supported by Stichting Fonds Dr. Catharine van Tussenbroek and 10.13039/501100003246Nederlandse Organisatie voor Wetenschappelijk Onderzoek, project number: Vici 16131.

## CRediT authorship contribution statement

**Mirjam Visscher**: Investigation, Software, Formal analysis, Writing – original draft. **Miguel A. Pleitez:** Investigation, Methodology, Writing – review & editing. **Kim Van Gaalen:** Resources. **Ingeborg M. Nieuwenhuizen-Bakker**: Resources. **Vasilis Ntziachristos:** Conceptualization. **Gijs van Soest:** Supervision, Writing – original draft, Writing – review & editing.

## Declaration of Competing Interest

The authors declare that they have no known competing financial interests or personal relationships that could have appeared to influence the work reported in this paper.
