# Supplementary material for: Label-free analytic histology of carotid atherosclerosis by mid-infrared optoacoustic microscopy
Source: Photoacoustics. 2022 Apr 11;26:100354. doi: 10.1016/j.pacs.2022.100354 (PMC9020099; doi:10.1016/j.pacs.2022.100354)
Supplement: Supplementary file 1 — Supplementary material [file mmc1.docx]

**Supplement to**

**Label-free analytic histology of carotid atherosclerosis by mid-infrared optoacoustic microscopy**

Mirjam Visscher^1^, Miguel A. Pleitez^2,3^, Kim Van Gaalen^1^, Ingeborg M. Nieuwenhuizen-Bakker^1^, Vasilis Ntziachristos^2,3^, Gijs Van Soest^1^

1. Department of Cardiology, Erasmus MC University Medical Center Rotterdam, PO Box 2040, 3000 CA Rotterdam, The Netherlands
2. Institute of Biological and Medical Imaging, Helmholtz Zentrum München, Neuherberg, Germany
3. Chair of Biological Imaging (CBI) and Center for Translational Cancer Research (TranslaTUM), Technische Universität München, München, Germany


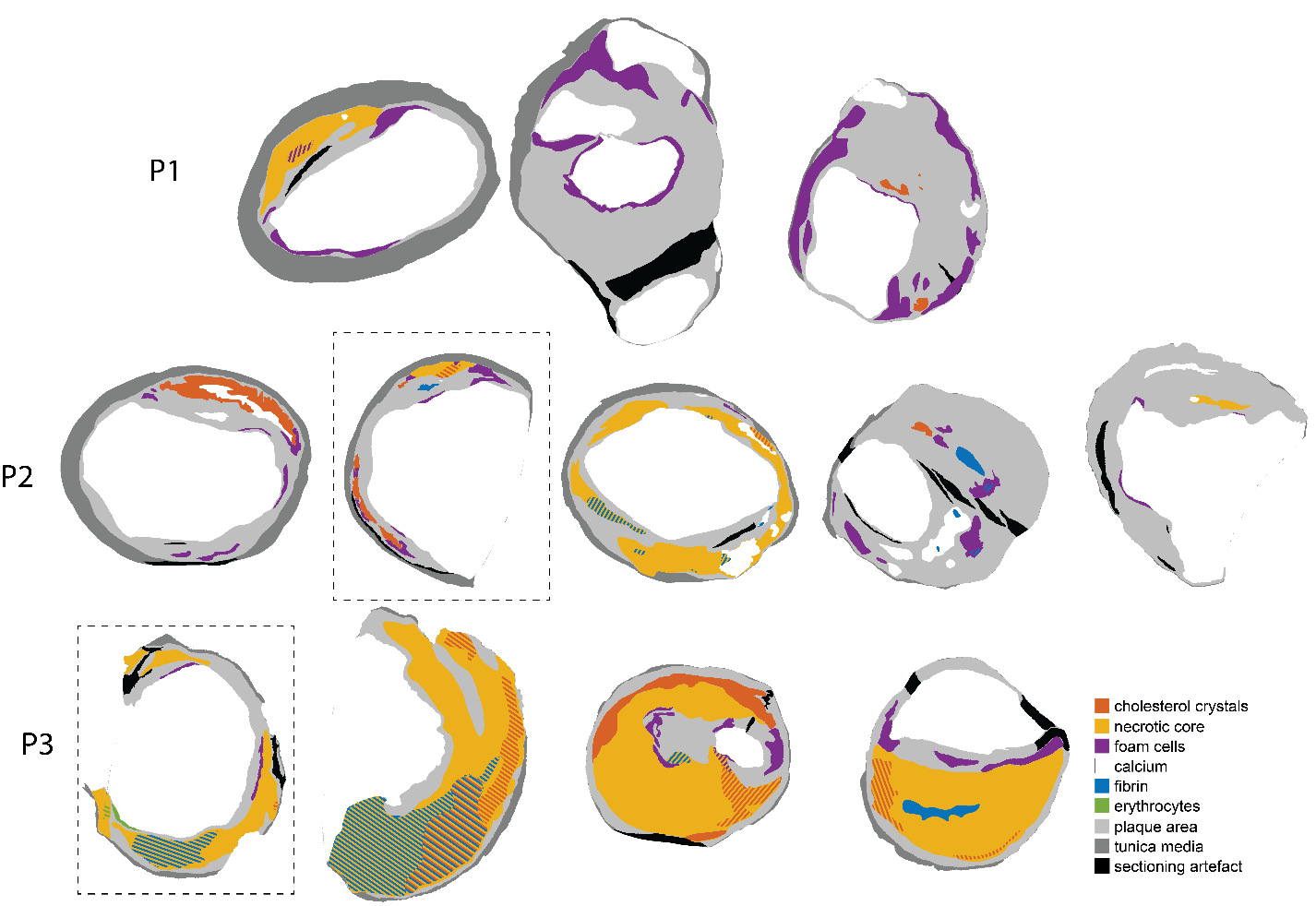


Supplementary figure S1: Histology analysis of all analyzed sections, grouped per patient. Boxed sections are not included in the histology comparison in Suppl. Fig. S2.


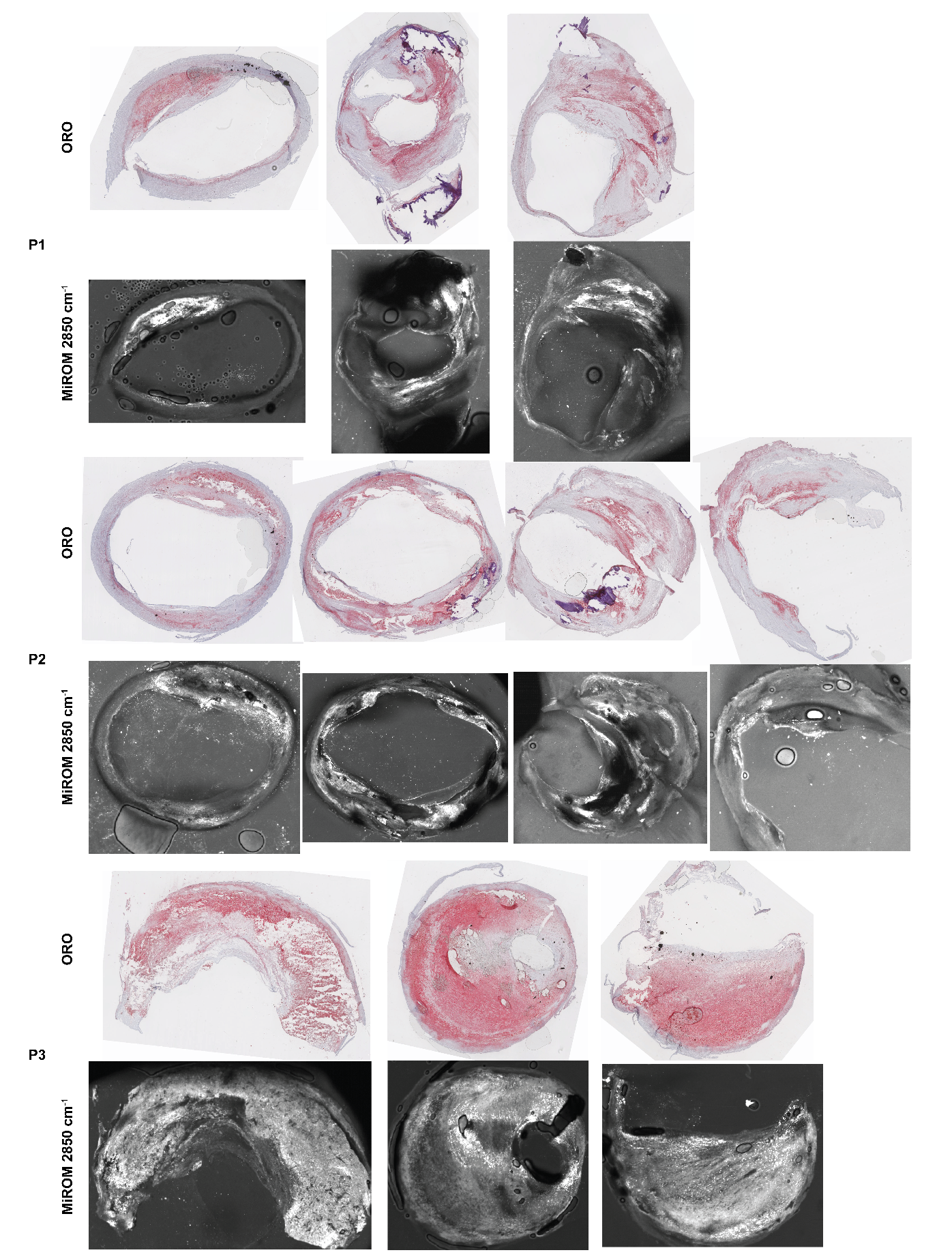


Supplementary figure S2: Overviews of the Oil Red O-stained sections compared to MiROM scans at 2850 cm-1. Two sections have been omitted from this comparison due to prominent bubble artifacts in the MiROM data.
